# Associations of Self-Reported Periodontal Disease With Metabolic Syndrome and Number of Self-Reported Chronic Conditions

**Published:** 2011-04-15

**Authors:** Lillian Bensley, Juliet VanEenwyk, Eric M. Ossiander

**Affiliations:** Washington State Department of Health, Office of Epidemiology; Washington State Department of Health, Olympia, Washington; Washington State Department of Health, Olympia, Washington

## Abstract

**Introduction:**

Increasing evidence supports associations between periodontal disease and various chronic conditions. Possible explanations include chronic inflammatory processes, shared pathogens, and shared risk factors, such as smoking and psychosocial stress. The objective of this study was to assess associations of periodontal disease with metabolic syndrome and number of chronic diseases.

**Methods:**

As part of the Washington Adult Health Survey, a household-based cross-sectional study conducted during 2006-2007 among adults aged 25 years or older in Washington State, we collected questionnaire data, blood samples, and anthropometric measures. We used these data to assess associations of periodontal disease with metabolic syndrome and the number of self-reported chronic diseases, controlling for age, sex, annual household income, smoking, and psychosocial stress. We used both complete case and multiple imputation Poisson regression analyses.

**Results:**

In the adjusted complete case analysis, 1.4 times as many chronic conditions were found among people with severe compared with no periodontal disease, and people with severe periodontal disease were 1.5 times more likely to have metabolic syndrome than people with no periodontal disease. Arthritis and liver disease were individually associated with severe periodontal disease. Results of the multiple imputation analyses were similar.

**Conclusion:**

These results suggest that people with severe periodontal disease are likely to have more chronic diseases and are more likely to have metabolic syndrome compared with people without periodontal disease. Research about the effectiveness of periodontal treatment to help prevent or control chronic diseases is needed.

## Introduction

Increasing evidence suggests that periodontal disease is associated with various chronic conditions. Particular attention has been paid to the possible role of periodontal disease in coronary heart disease (CHD) (1). The notion that improving periodontal health may reduce the risk of cardiovascular disease has public health implications because of the high prevalence of both of these diseases (2,3). A recent review provides evidence for the promise of this approach (4).

Several processes have been proposed through which periodontal disease may contribute to CHD or other chronic diseases. Systemic inflammation, arising in response to periodontal infection, may contribute to the initiation or progression of CHD (5) and other chronic diseases. A review of studies of periodontal disease and CHD notes that both the association of periodontal disease with C-reactive protein and other measures of systemic inflammation and the improvements in these measures following periodontal treatment support the notion that periodontal disease represents a chronic infection resulting in a chronic inflammatory state (1). In a related process, periodontal pathogens may infect other body systems, as evidenced by the well-established link between oral bacteria and infective endocarditis (2) and by studies that have identified DNA from periodontal pathogens in atherosclerotic plaques (4). Only an estimated 40% to 50% of the bacteria in the human oral cavity have been cultured, making our understanding of potential pathogens incomplete (6).

Shared risk factors may also contribute to an association between periodontal disease and other chronic diseases. Psychosocial stress and smoking are risk factors for both periodontal disease (5,7,8) and various respiratory and cardiovascular diseases (9,10). Psychosocial stress may adversely affect periodontal health and contribute to other chronic diseases either through immune suppression or behavioral changes (7). The breadth of the possible associations and the causal relationships are not well understood, and causal relationships may vary for different diseases.

Most of the research about the association between periodontal disease and CHD has examined the potential contribution of periodontal disease to CHD; in contrast, periodontal disease is considered a complication of diabetes. Rheumatoid arthritis may also adversely affect periodontal health (2). The inflammatory processes involved in these 3 diseases have been suggested as a likely link to periodontal disease. Periodontal disease may also contribute to difficulty with glucose control among diabetics. Less is known about a possible association between periodontal disease and liver disease. However, a recent study (11) found more urgent needs for periodontal treatment among 1999-2004 National Health and Nutrition Examination Survey (NHANES) participants who had arthritis, diabetes, a liver condition, or who had experienced a stroke.

Metabolic syndrome has been associated with periodontal disease. Low-grade systemic inflammation, originating in periodontal infection, may contribute to metabolic syndrome (12).

We know of no studies that have examined the risk of total chronic disease associated with having periodontal disease. We used data from the Washington Adult Health Survey (WAHS) to assess associations of periodontal disease with metabolic syndrome and the number of self-reported chronic diseases, controlling for age, sex, annual household income, smoking, and psychosocial stress.

## Methods

### Overview

WAHS was a field survey designed primarily to estimate the statewide prevalence of high blood pressure and high blood cholesterol and determine whether these differed for those living in households with an annual household income less than $35,000 compared with households with higher incomes. We included adults aged 25 years or older who spoke English or Spanish, lived in the sampled residence at least half the year, and were their own legal guardian. We excluded pregnant women and people with hemophilia or who were being treated for cancer. WAHS was conducted from August 2006 to November 2007.

WAHS used a 3-stage stratified cluster design. At the first stage we randomly selected census block groups from 3 strata defined by block group median income (<$25,000, $25,000-$34,999, and ≥$35,000). We oversampled from the lower income strata to increase the number of low-income respondents in the sample. We randomly selected housing units within block groups and randomly selected 1 adult aged 25 years or older from each housing unit. Potential participants received a home visit for recruitment and enrollment, and participants received additional home visits to collect interview information, a fasting blood sample, physical measurements, and for a check of medications. Participants also completed self-administered health and food frequency questionnaires. Participants received a $45 debit card and information about their blood pressure, blood glucose and lipids, and body mass index. The Washington State institutional review board approved all procedures. More detail about the methods is available on request. This study used information from the self-administered health questionnaire and the interview, data obtained from the physical measures, and clinical data from the blood analyses.

### Severity of self-reported periodontal disease

The measure of periodontal disease severity was constructed with 3 levels: severe, mild/moderate, or none. This measure was coded as *severe* if the participant answered yes to either the question "Have you ever had scaling, root planing, surgery, or other treatment for gum disease?" or "Have you ever had any teeth that have become loose by themselves without injury?" (yes, no, not sure). The measure was coded as *mild/moderate* if participants answered no to the questions about periodontal treatment and loose teeth but they either answered "fair" or "poor" to the question "How would you rate the health of your gums?" (excellent, very good, good, fair, or poor), or yes to the question "Has a dental professional ever told you that you have gum disease?" (yes, no, not sure). The measure was coded as *none* if participants answered "excellent," "very good," or "good" to the question about current gum health and no to the other questions.

### Metabolic syndrome

We used the American Heart Association definition (13) to determine whether participants had metabolic syndrome. A person was defined as having metabolic syndrome if they met at least 3 of the following criteria: 1) waist circumference equal to or greater than 102 cm (40 inches) for men or 88 cm (35 inches) for women, 2) fasting triglycerides equal to or greater than 150 mg/dL, 3) fasting high-density lipoprotein (HDL) cholesterol lower than 40 mg/dL for men or 50 mg/dL for women, 4) systolic blood pressure equal to or greater than 130 mm Hg or diastolic blood pressure equal to or greater than 85 mm Hg, or 5) fasting blood sugar equal to or greater than 100 mg/dL. A nurse drew, processed, and shipped the fasting blood sample according to protocols provided by the Northwest Lipid Metabolism and Diabetes Research Laboratories, which conducted the blood glucose and lipid analyses. The nurse measured blood pressure according to recommendations from the Subcommittee of Professional and Public Education of the American Heart Association Council on High Blood Pressure Research (14) and obtained the waist circumference measurement following NHANES protocols (15).

### Number of chronic conditions

The measure of chronic conditions was adapted from the NHANES 2003-2004 Medical Conditions Questionnaire, which provided interview data on a broad range of health conditions (15). The interviewer asked participants the stem question, "Has a doctor or other health professional ever told you that you have . . ." followed by "asthma; diabetes or 'sugar diabetes' (other than during pregnancy); congestive heart failure; coronary heart disease; angina, also called angina pectoris; a heart attack, also called myocardial infarction; a stroke; a transient ischemic attack, sometimes called TIA or 'mini-stroke'; emphysema; osteoporosis; poor circulation in legs or feet, also called peripheral vascular disease; chronic bronchitis; cancer or malignancy of any kind; any kind of liver condition; or arthritis?" We summed the number of yes responses, with the exceptions that 1) we did not include cancer because people taking cancer medications were excluded from the study and 2) we counted CHD as yes to any of the questions regarding CHD, angina, or heart attack; and stroke/TIA as yes to either stroke or TIA.

### Smoking

The measure of cigarette smoking was obtained from a Behavioral Risk Factor Surveillance System question (16) that was included on the self-administered questionnaire. Smoking was coded as *yes* if participants answered yes to the question "Have you smoked at least 100 cigarettes in your entire life?" (yes or no) and "every day" or "some days" in response to the question "Do you now smoke cigarettes every day, some days, or not at all?" Smoking was coded as *no* if participants answered no to the first question or "not at all" to the second question.

### Psychosocial stress

The measure of psychosocial stress was the Perceived Stress Scale (17), a 10-item scale designed to measure the degree to which situations in one's life are appraised as stressful (eg, "In the last month, how often have you felt that you were unable to control the important things in your life?" never = 0, almost never = 1, sometimes = 2, fairly often = 3, or very often = 4). Items were reverse-scored as necessary and summed.

### Data analysis

The major analyses were 2 Poisson regressions in which the outcome measures were metabolic syndrome (yes or no) and number of chronic conditions (range, 0-10), and the major predictor measure was periodontal disease (severe, mild/moderate, or none). For each analysis, the major predictor measure was entered, followed by the covariates. Covariates were age, sex, annual household income (<$35,000, $35,000-$50,000, or >$50,000), smoking (yes or no), and psychosocial stress scores. All analyses used SAS-callable SUDAAN to account for the clustered sampling design (SAS Institute, Inc, Cary, North Carolina). For the analysis of the number of chronic conditions we used Poisson regression because of the nonnormal distribution of the outcome variable. Metabolic syndrome is not a rare condition, and the odds ratios estimated by a logistic regression analysis would not be a good approximation to the prevalence ratio. Therefore, we also used Poisson regression for the metabolic syndrome analysis to estimate the prevalence ratio directly.

For each observation, we computed an analysis weight that was a combination of the inverse of the sampling probability and a poststratification weight to make the sample resemble the Washington State population in age and sex. All analyses incorporated the analysis weights.

Major analyses were complete case analyses, which used all of the cases with complete data for that analysis. The results of a complete case analysis can be biased if the missing data are not missing at random (18). Therefore we also conducted a multiple imputation analysis (19), which can provide valid results under the less stringent assumption that missingness may depend on either the outcome or the predictors but does not depend on unmeasured data after conditioning on the observed data. For the multiple imputation analyses, the only cases that were excluded were participants who were missing data on both the major predictor measure (periodontal disease severity) and the outcome measure for that analysis, based on the reasoning that these cases did not bring information to the analysis. These constituted no more than 1% to 2% of the cases.

## Results

### Response rate and missing data

After determining eligibility, we recruited 1,534 people, and of these, 672 (44%) participated. The Council of American Survey Research Organizations (CASRO) (20) response rate, which also takes into account people we could not reach or for whom we could not determine eligibility, was 38%. The CASRO response rate is the product of 2 other rates: 1) the screening rate, which is the proportion of households that complete the screening process; and 2) the cooperation rate, which is the proportion of screened eligible households that participated in the survey. We estimated the CASRO response rate to be higher in the lowest income stratum (45%) than in the middle (39%) or highest (34%) income strata.

Of the 672 participants, 456 had complete data for all of the analyses. The remainder were missing at least 1 measure for at least 1 analysis: 10% of the sample had missing data on the measure of periodontal health, 6% on the number of chronic diseases, 11% on metabolic syndrome, and 11% on at least 1 of the covariates.

### Sample characteristics

The sample was primarily non-Hispanic white, as is the Washington population (Table 1). The sample contained more women than men, but the poststratification weighting made the weighted percentages approximately equal. Oversampling low-income block groups led to relatively large numbers of low-income participants. However, weighted percentages of these measures are similar to the state as a whole. Approximately one-quarter of the participants reported severe periodontal disease, approximately one-fifth reported mild/moderate disease, and more than half reported no history of periodontal disease.

### Risk for chronic disease associated with periodontal disease

Both the complete case and multiple imputation analyses showed more chronic conditions among people with severe periodontal disease, and this association remained significant after adding the covariates (Table 2). In the adjusted complete case analysis, 1.4 times as many chronic conditions were reported among people with severe compared with no periodontal disease. Age, smoking, and psychosocial stress also were significantly associated with the number of chronic conditions in both analyses (data not shown).

### Risk for metabolic syndrome associated with periodontal disease

Both the complete case and multiple imputation analyses showed more risk for metabolic syndrome among people with severe periodontal disease, and this association remained significant after adding the covariates (Table 2). In the adjusted complete case analysis, participants with severe periodontal disease were 1.5 times more likely to have metabolic syndrome compared with participants without periodontal disease. Age and sex also were significantly associated with risk for metabolic syndrome in the complete case analysis; in the multiple imputation analysis, age but not sex achieved significance (data not shown).

### Risk for individual chronic conditions associated with periodontal disease

Both the complete case and multiple imputation analyses showed more risk for liver disease and arthritis among people with severe periodontal disease, and these associations remained significant after adding the covariates (Table 3).

## Discussion

Participants who reported having severe periodontal disease reported approximately 40% more chronic conditions than participants who reported having no periodontal disease. To our knowledge, this study is the first to estimate the increased risk of overall chronic illness associated with periodontal disease. The fact that periodontal disease, as a risk factor, is not specific to a single disease but appears to be associated with varied chronic conditions is consistent with the concept that it may contribute to inflammation and damage to various systems. Other persistent, chronic, or recurrent infections may also play a role, as may the total burden of infection.

Our results are similar to a study of NHANES participants (11), which found associations between need for periodontal treatment and self-reported arthritis, diabetes, a liver condition, and having had a stroke. However, our ability to identify associations of periodontal disease with specific conditions was limited by small numbers. We did not find the expected association between periodontal disease and CHD; based on 3 meta-analyses including many thousands of participants (1,21,22), this association may be in the range of a 15% to 30% increase in risk, and our study did not have the power to detect an association of this size. Small numbers may also have reduced our ability to identify a link between severe periodontal disease and diabetes, a comparison which achieved significance in our study before, but not after, covariates were added to the model.

Major factors potentially limiting the validity of this research are the low response rate and the use of self-reported measures of periodontal disease and chronic disease. Although some large-scale studies, notably NHANES (15), have included periodontal clinical examinations, the expense of conducting clinical exams has led to efforts to develop and use self-reported measures for surveillance and research. A Centers for Disease Control and Prevention and American Academy of Periodontology workgroup concluded that multivariable modeling of self-reported measures is promising for predicting the population prevalence of periodontitis (23). The specific measure used in this report has not been validated by clinical measures such as pocket depth but combines questions that have been identified as having good validity (24) or as contributing to multivariable models (25). More research is needed to validate the specific approach used in this research and to determine the optimal approach (in terms of validity, reliability, and cost effectiveness) to self-reported periodontal disease.

Second, the response rate for this study (CASRO response rate 38%) was low, so we compared characteristics of the WAHS sample with the American Community Survey (ACS, 26). These characteristics were similar on most measures. Although a small number of characteristics (such as marital status) differed between WAHS and ACS, both number of diagnoses and metabolic syndrome remained significant when these were added as covariates, so these differences do not appear to have affected the major results. Furthermore, several recent reviews indicate that there is no consistent relationship between response rates and the amount of nonresponse bias. The range of response rates in the studies they reviewed was about 25% to 85% (27-29).

The sample was designed to be representative of the Washington State population. However, unless the associations of periodontal disease with chronic disease and metabolic syndrome vary between populations, the results may be generalized to adults in other states.

The range of chronic conditions associated with periodontal disease suggests that interventions to increase periodontal health may have far-reaching effects on public health. A review by Tonetti (4) found that intensive periodontal therapy resulted in a decrease in systemic inflammation and an improvement of endothelial dysfunction in otherwise healthy subjects. Also, a recent study examining the medical costs of diabetes patients found a cost savings in the range of 3% to 8% for patients who were receiving regular dental care compared with those not receiving any preventive or periodontal services (30).

In conclusion, these results provide evidence that people with severe periodontal disease are more likely to have metabolic syndrome and other chronic conditions compared with people without periodontal disease. These associations did not appear to result from confounding from age, sex, income, smoking, or psychosocial stress. Intervention research about the effectiveness of periodontal treatment to prevent or control various chronic diseases, which have in common an inflammatory process, is needed.

## Figures and Tables

**Table 1:** Characteristics of Sample Participants in the Washington Adult Health Survey (N = 672), Washington State, 2006-2007

**Characteristic^a^**	No. (%)^b^
**Sex**	
Female	393 (51)
Male	279 (49)
**Race/ethnicity**	
Non-Hispanic white	497 (78)
Non-Hispanic black	29 (4)
Non-Hispanic Asian/Pacific Islander	37 (7)
Non-Hispanic American Indian/Alaska Native	11 (1)
Hispanic	91 (9)
**Education**	
High school graduate or less	244 (30)
Some college or technical school	242 (37)
College graduate or more	182 (32)
**Annual household income, $**	
<35,000	283 (28)
35,000-50,000	125 (20)
>50,000	226 (52)
**Periodontal disease severity**	
Severe	163 (24)
Mild/moderate	119 (18)
None	321 (58)
**Metabolic syndrome**	
No	217 (36)
Yes	384 (64)
**Smoke**	
No	518 (82)
Yes	146 (18)
**Arthritis**	
Yes	174 (23)
No	487 (77)
**Asthma**	
Yes	115 (17)
No	556 (83)
**Chronic bronchitis**	
Yes	34 (3)
No	632 (97)
**Congestive heart failure**	
Yes	14 (2)
No	656 (98)
**Coronary heart disease**	
Yes	44 (7)
No	625 (93)
**Diabetes**	
Yes	81 (10)
No	590 (90)
**Emphysema**	
Yes	11 (2)
No	658 (98)
**Liver disease**	
Yes	30 (4)
No	639 (96)
**Osteoporosis**	
Yes	50 (6)
No	615 (94)
**Peripheral vascular disease**	
Yes	46 (5)
No	614 (95)
**Stroke/transient ischemic attack**	
Yes	30 (4)
No	637 (96)

a Median age of participants was 48 years (range, 25 y to ≥90 y); people were asked to report their year of age up to 89 years, and ages 90 years or older were coded as "90 or older." The mean number of self-reported, physician-diagnosed conditions was 0.8 (range, 0-10), based on the following conditions: arthritis, asthma, chronic bronchitis, congestive heart failure, coronary heart disease, diabetes, emphysema, liver disease, osteoporosis, peripheral vascular disease, and stroke or transient ischemic attack. The median psychosocial stress score of participants was 13 (range, 0-39).

b Values for n may not sum to total because of missing data. Percentages are weighted.

**Table 2:** Associations of Self-Reported Severity of Periodontal Disease With Number of Self-Reported Chronic Conditions and Metabolic Syndrome, Washington Adult Health Survey (N = 672), Washington State, 2006-2007

Periodontal Disease History	n^a^	Mean No. of Chronic Conditions^b^	Ratio of the No. of Chronic Conditions	

			Crude Ratio (95% CI)	Adjusted Ratio^c^ (95% CI)
**Complete case analysis**				
None	278	0.6	1 [Reference]	1 [Reference]
Mild/moderate	101	0.8	1.2 (0.8-1.9)	1.3 (0.9-1.9)
Severe	128	1.2	1.9 (1.3-2.7)	1.4 (1.02-1.9)
**Multiple imputation analysis**				
None	341	0.7	1 [Reference]	1 [Reference]
Mild/moderate	141	0.9	1.2 (0.8-2.3)	1.4 (1.1-1.8)
Severe	181	1.2	1.6 (1.1-2.3)	1.3 (1.001-1.7)

**Periodontal Disease History**	**n^a^**	**% With Metabolic Syndrome^d^**	**Prevalence Ratio for Metabolic Syndrome**	

			**Crude Ratio (95% CI)**	**Adjusted Ratio (95% CI)**

**Complete case analysis**				
None	264	30	1 [Reference]	1 [Reference]
Mild/moderate	95	33	1.1 (0.8-1.6)	1.2 (0.8-1.8)
Severe	122	50	1.7 (1.2-2.3)	1.5 (1.2-2.1)
**Multiple imputation analysis**				
None	342	33	1 [Reference]	1 [Reference]
Mild/moderate	137	36	1.1 (0.8-1.5)	1.1 (0.8-1.6)
Severe	181	48	1.5 (1.1-1.9)	1.3 (1.1-1.7)

Abbreviation: CI, confidence interval.

a The complete case analyses excluded participants who had missing data on any of the variables in that analysis, and the multiple imputation analyses excluded participants who had missing data on both the predictor and outcome variables in that analysis.

b Means are weighted.

c Adjusted for sex, age, annual household income, smoking, and psychosocial stress.

d Percentages are weighted.

**Table 3:** Associations of Self-Reported Severity of Periodontal Disease With Individual Chronic Conditions, Washington Adult Health Survey (N = 672), Washington State, 2006-2007

Chronic Condition^a^	n^b^	% With the Condition^c^	Prevalence Ratio	
			Crude Ratio(95% CI)	Adjusted Ratio^d^ (95% CI)
**Complete case analyses**				
Arthritis	527	23	1.8 (1.2-2.9)	1.5 (1.1-2.2)
Asthma	536	17	1.3 (0.8-2.2)	1.2 (0.7-2.0)
Chronic bronchitis	532	3	2.0 (0.5-7.6)	1.0 (0.3-3.1)
Congestive heart failure	535	2	5.6 (0.8-38.0)	4.4 (0.4-50.6)
Coronary heart disease	534	6	1.2 (0.4-3.67)	1.0 (0.3-3.4)
Diabetes	535	10	2.3 (1.2-4.8)	1.7 (0.9-3.2)
Emphysema	533	2	1.4 (0.2-8.9)	1.7 (0.3-10.7)
Liver disease	534	4	6.6 (2.0-22.4)	5.7 (1.7-19.4)
Osteoporosis	530	5	1.4 (0.4-5.7)	1.2 (0.4-3.6)
Peripheral vascular disease	527	4	1.6 (0.5-5.1)	0.9 (0.2-3.5)
Stroke or transient ischemic attack	532	3	2.5 (0.7-9.3)	1.5 (0.4-5.1)
**Multiple imputation analyses**				
Arthritis	670	24	1.6 (1.1-2.5)	1.5 (1.02-2.2)
Asthma	672	17	1.2 (0.7-2.1)	1.1 (0.6-1.9)
Chronic bronchitis	671	4	1.8 (0.6-5.8)	1.1 (0.4-3.0)
Congestive heart failure	671	2	2.3 (0.6-9.0)	1.7 (0.4-7.6)
Coronary heart disease	671	7	1.1 (0.4-2.9)	0.9 (0.3-2.6)
Diabetes	672	10	1.8 (0.9-3.6)	1.4 (0.8-2.7)
Emphysema	672	2	1.1 (0.2-5.8)	1.1 (0.2-6.5)
Liver disease	671	4	3.0 (1.3-6.9)	2.7 (1.2-6.2)
Osteoporosis	672	6	1.2 (0.4-3.9)	1.2 (0.6-2.6)
Peripheral vascular disease	670	6	1.5 (0.7-3.4)	1.0 (0.4-2.5)
Stroke or transient ischemic attack	671	4	1.8 (0.6-5.3)	1.3 (0.4-3.7)

Abbreviation: CI, confidence interval.

a Self-reported physician-diagnosed conditions.

b The complete case analyses excluded participants who had missing data on any of the variables in that analysis, and the multiple imputation analyses excluded participants who had missing data on both the predictor and outcome variables in that analysis.

c Percentages are weighted.

d Adjusted for sex, age, annual household income, smoking, and psychosocial stress.
